# Supplementary material for: A stapled lipopeptide platform for preventing and treating highly pathogenic viruses of pandemic potential
Source: Nat Commun. 2024 Jan 4;15:274. doi: 10.1038/s41467-023-44361-1 (PMC10766962; doi:10.1038/s41467-023-44361-1)
Supplement: Supplementary file 1 — Supplementary Information [file 41467_2023_44361_MOESM1_ESM.pdf]

A Stapled Lipopeptide Platform for Preventing and Treating Highly Pathogenic Viruses of Pandemic Potential

Gregory H. Bird, J.J. Patten, William Zavadoski, Nicole Barucci, Marina Godes, Benjamin M. Moyer, Callum D. Owen, Paul DaSilva-Jardine, Donna Neuberg, Richard A. Bowen, Robert A. Davey, and Loren D. Walensky

Supplementary Information

Supplementary Figures and Legends 1-9

Supplementary Table 1

a

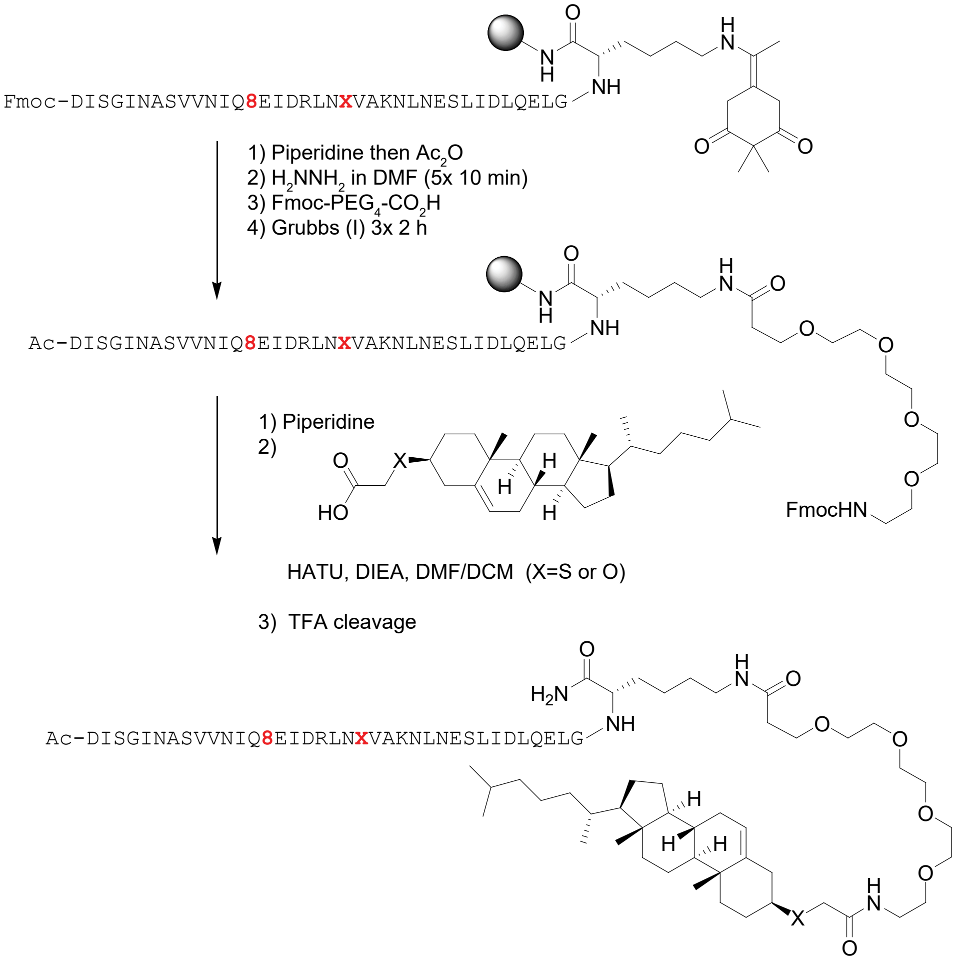

b

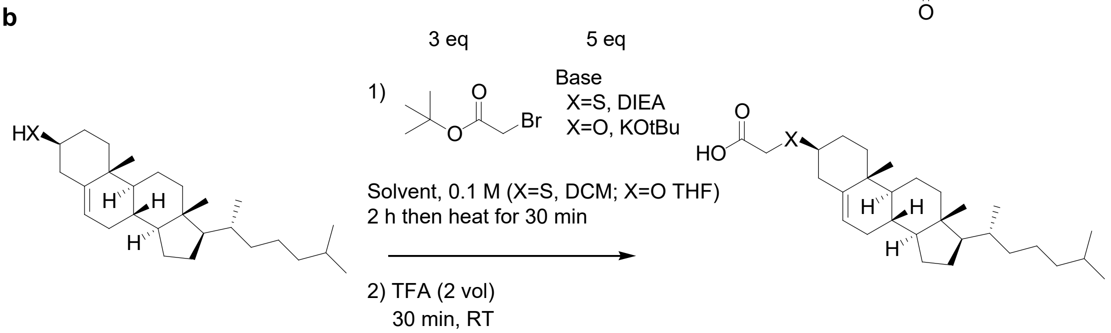

Bird et al. Figure S1

Supplementary Fig. 1 Synthetic schema for production of stapled lipopeptides. a, Synthetic route for on-resin derivatization of the nascent peptide with a PEG linker, followed by olefin metathesis, and sterol conjugation to generate a stapled lipopeptide of the SARS-CoV-2 HR2 domain. **b**, Synthetic route for production of 2-(sterol)acetic acid used for on-resin lipidation of the stapled HR2 peptide.

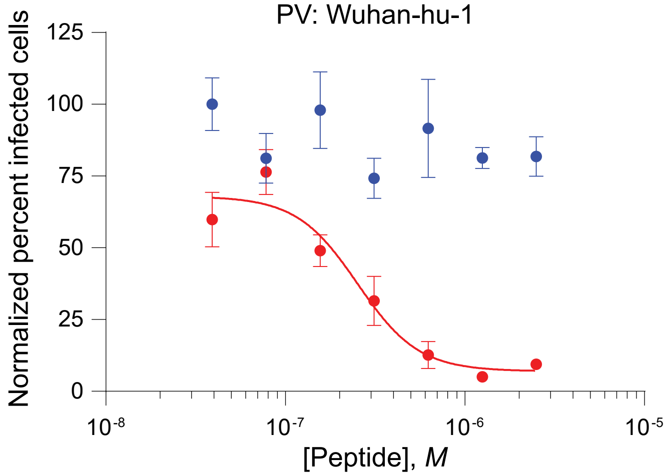

	IC_{50}	95% CI
HR2 (aa 1168-1205)	N/A	N/A
HR2-PEG_4-TC	2.5×10^{-7}	1.6×10^{-7} - 3.9×10^{-7}

Bird et al. Figure S2

Supplementary Fig. 2 Comparative antiviral activity of the native HR2 domain and a thiocholesterol derivative. a, A native HR2 peptide (amino acids 1168-1205) and PEG_4-thiocholesterol derivative were tested in an infectivity assay using the Wuhan-Hu-1 pseudovirus. Data are mean ± SEM for assays performed in technical quadruplicate and then repeated with similar results. IC_{50} values were calculated by nonlinear regression analysis of the dose-response curves.

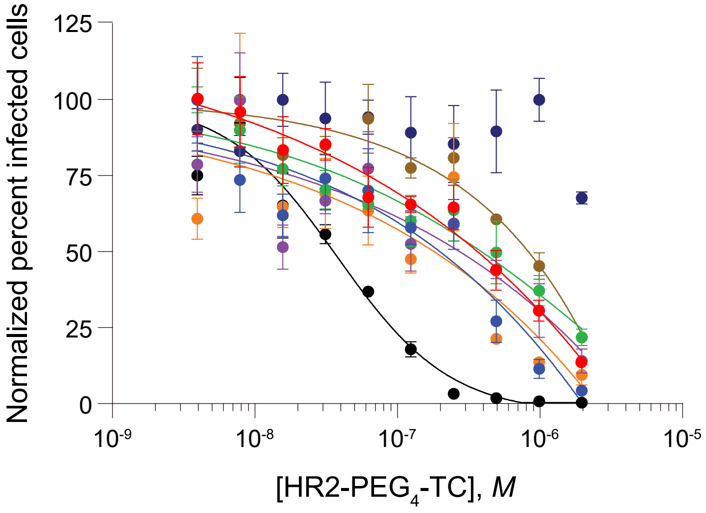

	IC_{50}	95% CI
Wuhan-hu-1	1.6×10^{-7}	6.2×10^{-8} - 2.4×10^{-7}
B.1 D614G	2.6×10^{-7}	1.1×10^{-7} - 3.6×10^{-7}
B.1.1.7	3.4×10^{-7}	1.1×10^{-7} - 5.7×10^{-7}
B.1.351	2.1×10^{-7}	1.8×10^{-7} - 4.4×10^{-7}
B.1.617.2	1.3×10^{-7}	3.3×10^{-8} - 2.8×10^{-7}
B.1.1.529	3.5×10^{-8}	2.7×10^{-8} - 4.4×10^{-8}
SARS-CoV (Urbani)	7.3×10^{-7}	3.8×10^{-7} - 1.1×10^{-6}
VSV with MLV core	N/A	N/A

Bird et al. Figure S3

Supplementary Fig. 3 Anti-pseudoviral activity of an unstapled lipopeptide of the SARS-CoV-2 HR2 domain. Dose-titration of the unstapled HR2-PEG_4-thiochol peptide in a panel of pseudoviral assays, which included Wuhan-Hu-1, D614G, B.1.1.7 (Alpha), B.1.351 (Beta), B.1.617.2 (Delta), B.1.1.529.1 (Omicron), SARS-CoV (Urbani), and VSV-G as a specificity-of-action control. Data are mean ± SEM for assays performed in technical quadruplicate and then repeated with similar results. IC_{50} values were calculated by nonlinear regression analysis of the dose-response curves.

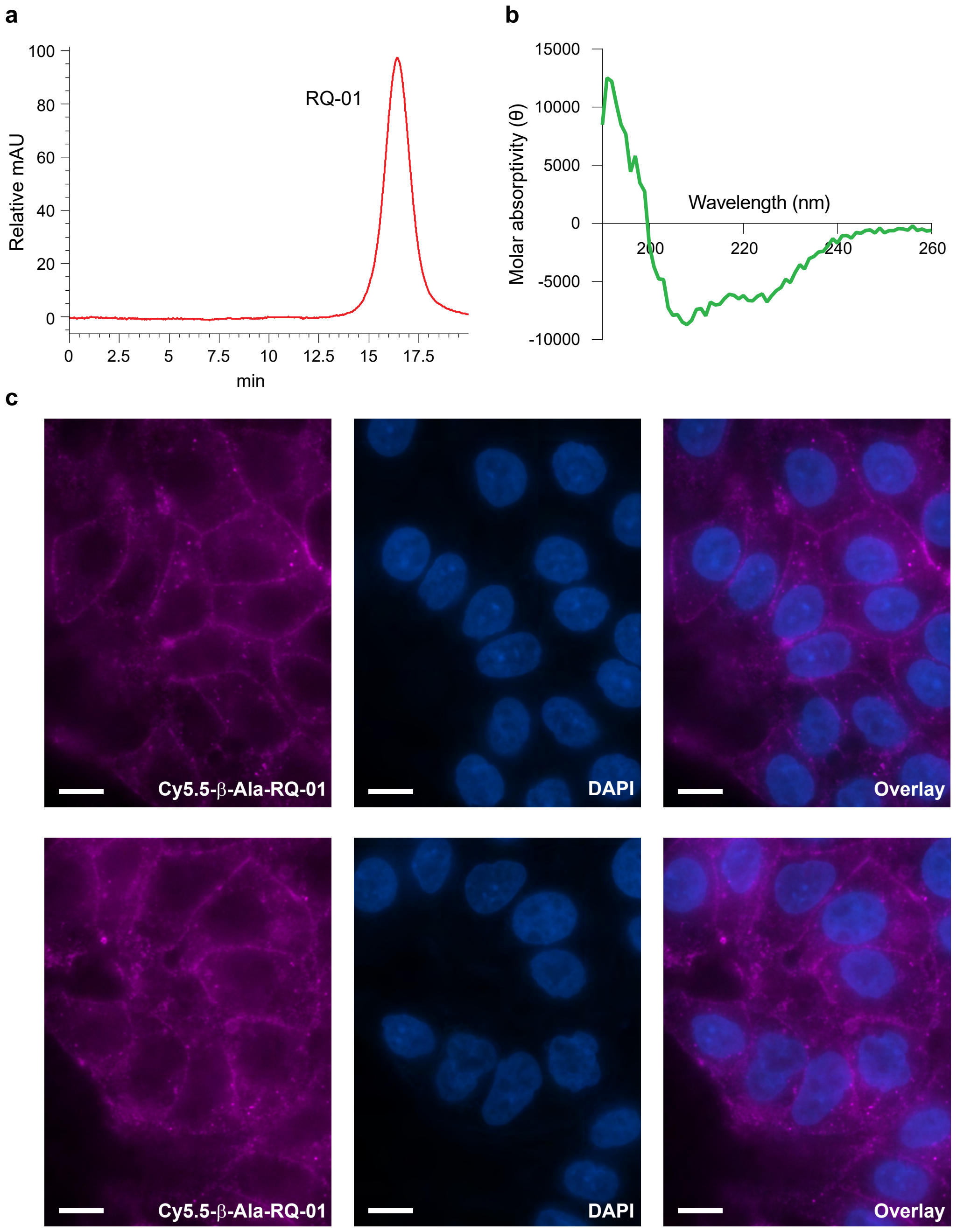

Bird et al. Figure S4

Supplementary Fig. 4 Homogeneity, α-helicity, and cellular imaging of RQ-01. a, RQ-01 eluted as a monodispersed peak by FPLC. **b**, RQ-01 exhibited α-helical character by circular dichroism. **c**, Fluorescence imaging of ACE2-A549 cells treated with Cy5.5-β-Ala-RQ-01 (10 μM), demonstrating prominent plasma membrane and endosomal localizations. Scale bar, 10 μm

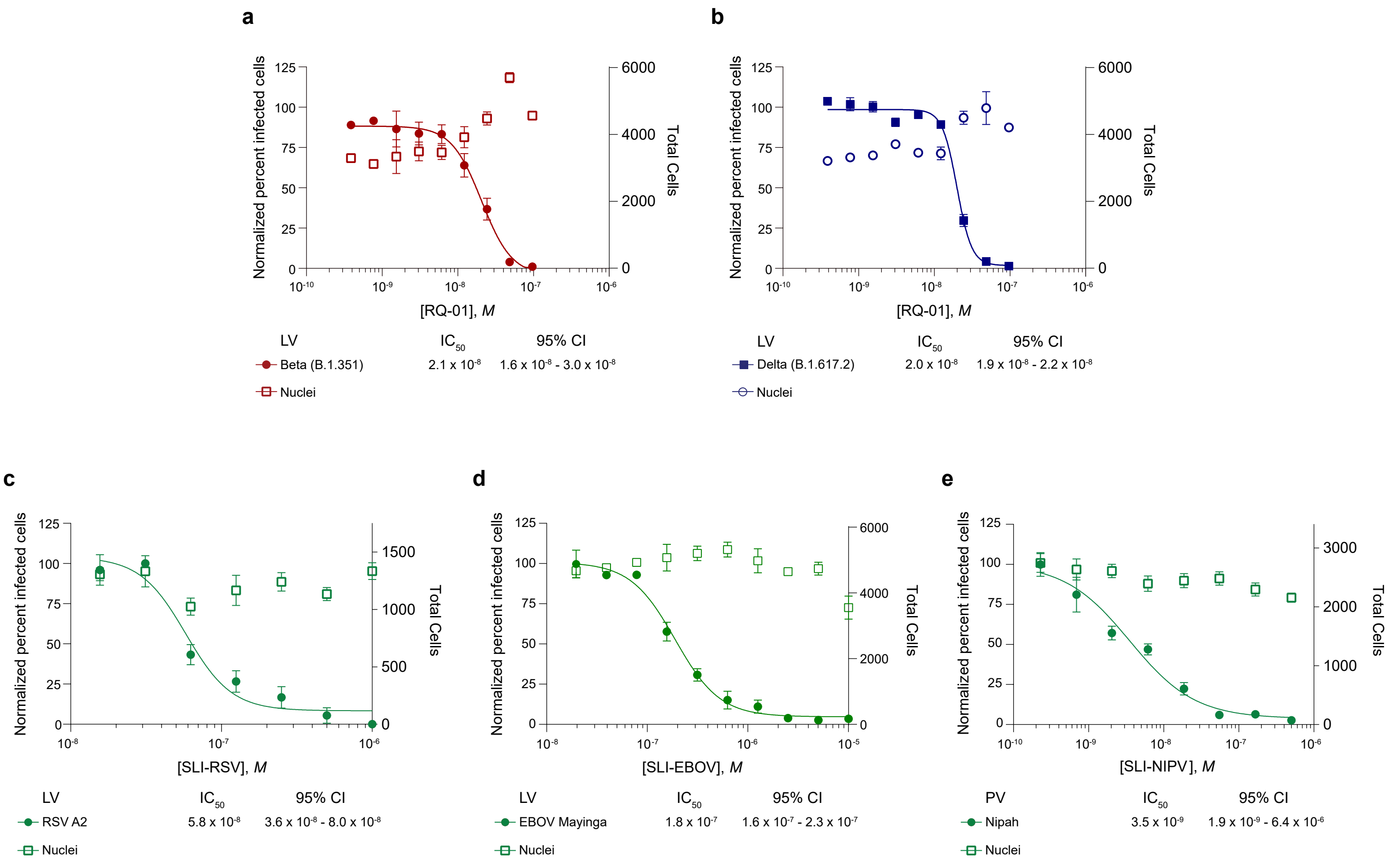

Bird et al. Figure S5

Supplementary Fig. 5 Stapled lipopeptide inhibitors demonstrate potent antiviral activity without cytotoxicity. a-b, RQ-01 was tested in live virus assays using the SARS-CoV-2 Beta (B.1.351) and Delta (B.1.617.2) strains and percent infected cells and A549-ACE2 cell count were plotted. Data are mean ± SEM for assays performed in technical triplicate and then repeated with similar results. IC_{50} values were calculated by nonlinear regression analysis of the dose-response curves. **c-e**, Stapled lipopeptide inhibitors of RSV, Ebola, and Nipah were respectively tested against RSV A2 live virus (c), Ebola Mayinga live virus (d), and Nipah pseudovirus (e), and percent infected cells and A549, HeLa, and 293T cell counts, respectively, were plotted. Data are mean ± SEM for assays performed in at least technical triplicate (RSV, n=4; Ebola, n=3; Nipah, n=10) and then repeated with similar results. IC_{50} values were calculated by nonlinear regression analysis of the dose-response curves.

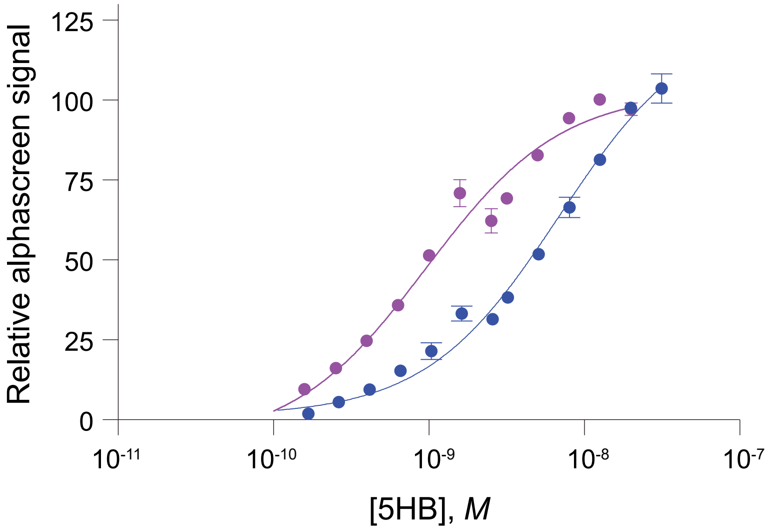

	EC_{50}	95% CI
HR2 (- staple, - linker-lipid)	6.8 x 10^{-9}	5.5 x 10^{-9} - 8.5 x 10^{-9}
RQ-01 (- linker-lipid)	0.9 x 10^{-9}	0.7 x 10^{-9} - 1.2 x 10^{-9}

Bird et al. Figure S6

Supplementary Fig. 6 Comparative 5-HB binding activity of a stapled and unstapled SARS-CoV-2 HR2 domain peptide. AlphaScreen assay demonstrating the binding activity of the RQ-01 stapled HR2 sequence (without linker-lipid attachment) and its unstapled analog, for the 5-helix bundle (5-HB) of SARS-CoV-2 comprised of 3 HR1 and 2 HR2 domains. Data are mean ± SEM for assays performed in technical quadruplicate and then repeated with similar results. IC_{50} values were calculated by nonlinear regression analysis of the dose-response curves.

RQ-01 $SARS_{HRC}$-PEG_4-chol

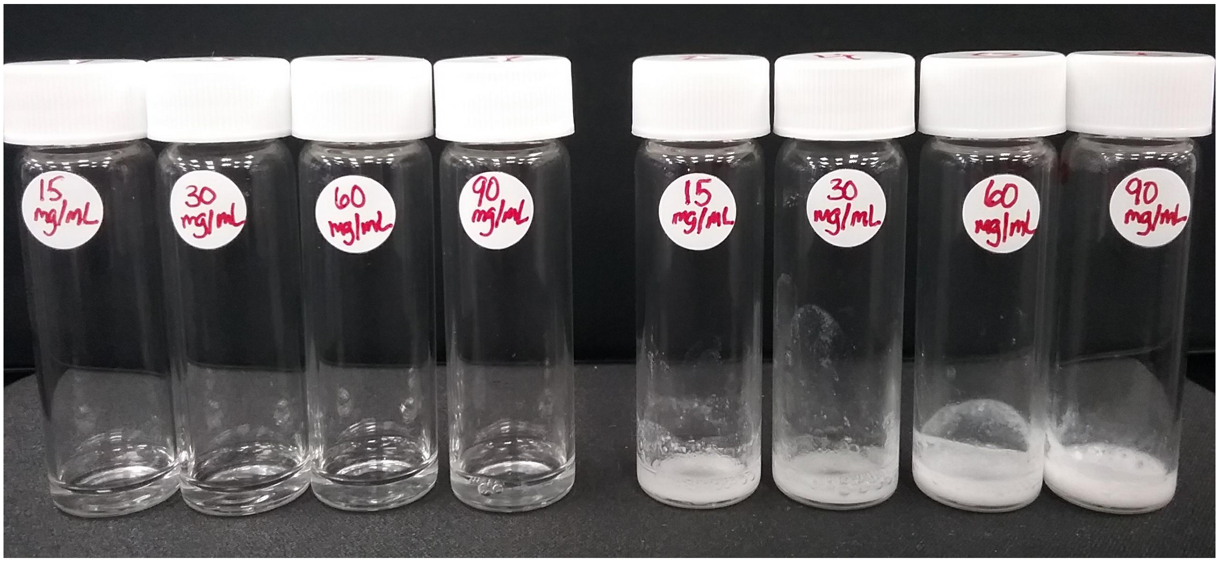

Bird et al. Figure S7

Supplementary Fig. 7 Comparative aqueous solubility of a stapled and unstapled lipopeptide inhibitor of SARS-CoV-2. RQ-01 and $SARS_{HRC}$-PEG_4-chol were each mixed with 50 mM sodium phosphate buffer, pH 7.0 and sonicated for 30 min at room temperature in order to achieve 15, 30, 60, and 90 mg/mL solutions. Whereas all RQ-01 solutions were clear, $SARS_{HRC}$-PEG_4-chol was insoluble across the range of concentrations.

a

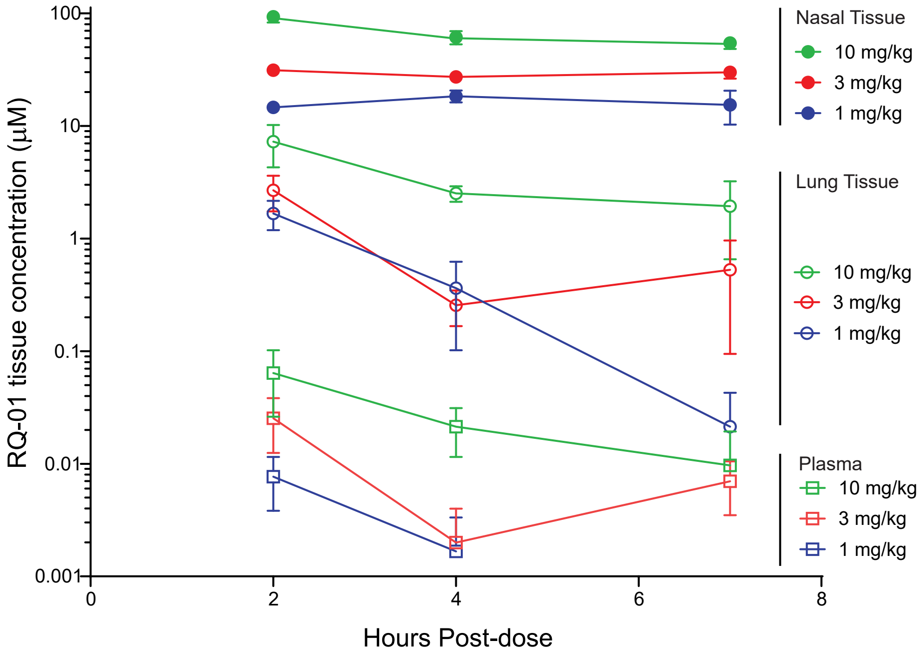

b

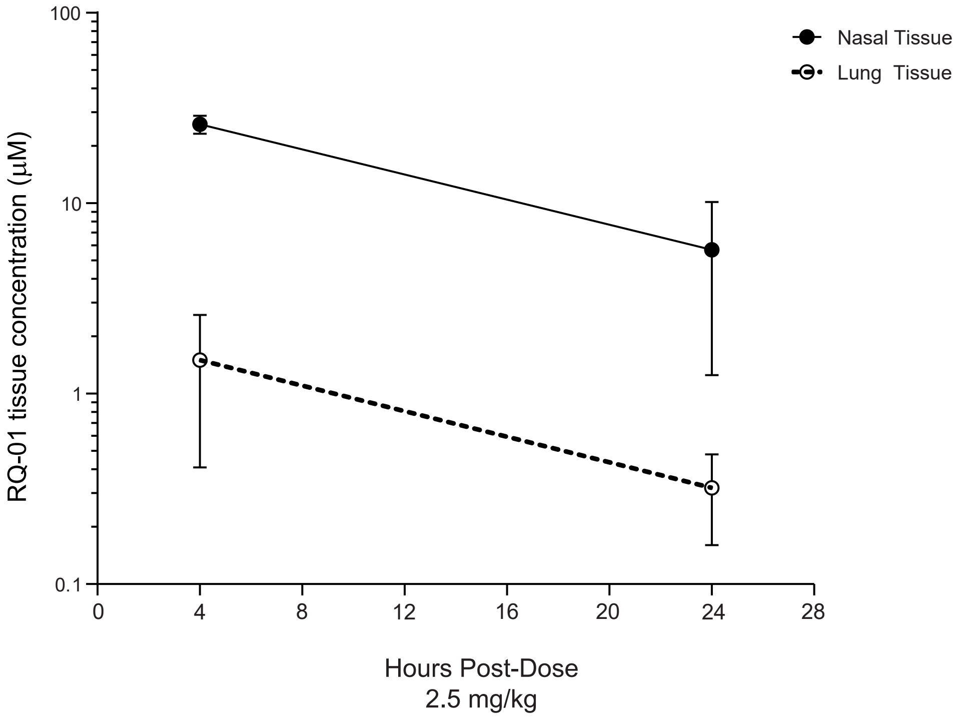

Bird et al. Figure S8

Supplementary Fig. 8 Tissue pharmacokinetic assessments of RQ-01 in mice and hamsters. **a**, Mice were treated intranasally with 1, 3, or 10 mg/kg of RQ-01 formulated in 0.1% HPMC/50 mM sodium phosphate, pH 6.5 and concentrations in nasal tissue, lung tissue, and plasma at 2, 4, and 7 hours after dosing, as detected by LC/MS-MS, were plotted. Data are mean ± SEM for RQ-01 levels quantified for n=3 mice per dosing level and time point. **b**, Hamsters were treated intranasally with a 2.5 mg/kg dose of RQ-01 formulated in 0.1% HPMC/50 mM sodium phosphate, pH 6.5 and concentrations in nasal and lung tissues at 4 and 24 hours after dosing, as detected by LC/MS-MS, were plotted. Data are mean ± SEM for RQ-01 levels quantified for n=2 hamsters per dosing level and time point.

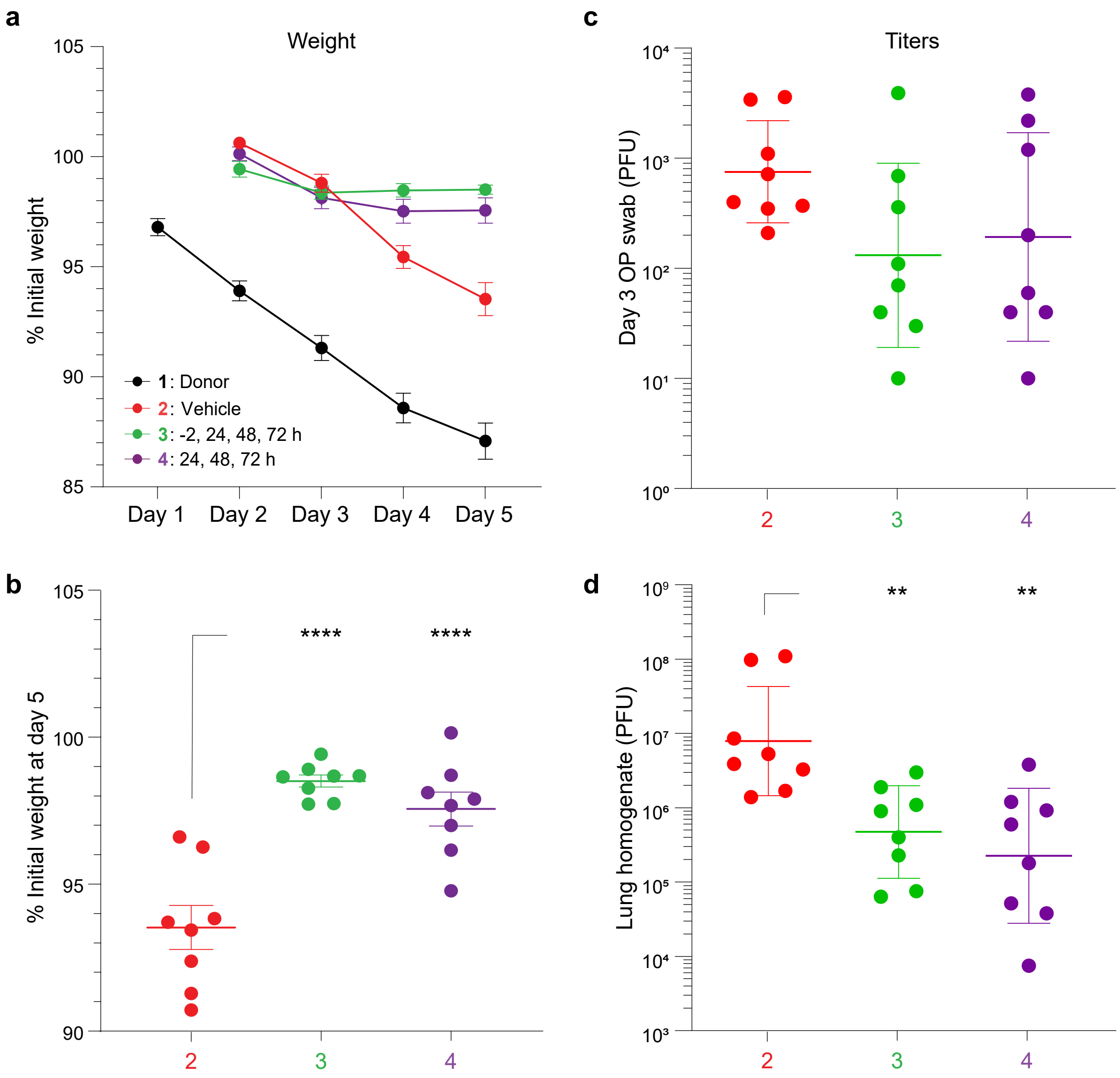

Bird et al. Figure S9

Supplementary Fig. 9 *In vivo* efficacy of RQ-01 in a hamster model of SARS-CoV-2 transmission. Male sentinel hamsters were treated with vehicle or 3 mg/kg RQ-01 at either -2, 24, 48, 72 h or 24, 48, 72 h relative to exposure to a donor animal. Plotted comparative outcome measures include daily percent initial weight (Day 0 for donors, Day 1 for sentinels) (**a**), percent initial weight on Day 5 (Groups 3 and 4, $p<0.0001$) (**b**), oropharyngeal (OP) swab titer from Day 3 (Group 3, $p=0.11$; Group 4, $p=0.24$) (**c**), and lung viral titers from Day 5 (Group 3, $p=0.008$; Group 4, $p=0.001$) (**d**). For outcomes measured in n=8 mice per treatment arm, data are mean ± SEM (**a-b**) and geometric mean ± geometric SD (**c-d**). One way ANOVA with Dunnett's Multiple Comparisons Test to Vehicle: ****, $p<0.0001$; ***, $p<0.001$; **, $p<0.01$; *, $p<0.05$

Peptide Name	SARS-CoV2 S (a.a. 1168-1205)	N-term	Staple location	PEG length	Sterol	Figure
HR2-PEG4-TC (SARS-CoV-2)	DISGINASVVNIQKEIDRLNEVAKNLNESLIDLQELG(K*)	Ac-	-	4	thiocholesterol	1d-h, 2g, S2, S3
SAH-HR2-A-PEG4-TC	DISGINASVV8IQKEIDXLNEVAKNLNESLIDLQELG(K*)	Ac-	A	4	thiocholesterol	1d-h
SAH-HR2-B-PEG4-TC	DISGINASVVN8QKEIDRXNEVAKNLNESLIDLQELG(K*)	Ac-	B	4	thiocholesterol	1d-h
SAH-HR2-C-PEG4-TC	DISGINASVVNI8KEIDRLXEVAKNLNESLIDLQELG(K*)	Ac-	C	4	thiocholesterol	1d-h
SAH-HR2-D-PEG4-TC	DISGINASVVNIQ8EIDRLNXVAKNLNESLIDLQELG(K*)	Ac-	D	4	thiocholesterol	1d- i, 2a-c, 2h
SAH-HR2-E-PEG4-TC	DISGINASVVNIQK8IDRLNEXAKNLNESLIDLQELG(K*)	Ac-	E	4	thiocholesterol	1d-h
SAH-HR2-F-PEG4-TC	DISGINASVVNIQKE8DRLNEVXKNLNESLIDLQELG(K*)	Ac-	F	4	thiocholesterol	1d-h
SAH-HR2-G-PEG4-TC	DISGINASVVNIQKEI8RLNEVAXNLNESLIDLQELG(K*)	Ac-	G	4	thiocholesterol	1d-h
SAH-HR2-H-PEG4-TC	DISGINASVVNIQKEID8LNEVAKXLNESLIDLQELG(K*)	Ac-	H	4	thiocholesterol	1d-h
SAH-HR2-I-PEG4-TC	DISGINASVVNIQKEIDR8NEVAKNXNESLIDLQELG(K*)	Ac-	I	4	thiocholesterol	1d-h
SAH-HR2-J-PEG4-TC	DISGINASVVNIQKEIDRL8EVAKNLXESLIDLQELG(K*)	Ac-	J	4	thiocholesterol	1d-h
SAH-HR2-K-PEG4-TC	DISGINASVVNIQKEIDRLN8VAKNLNXSLIDLQELG(K*)	Ac-	K	4	thiocholesterol	1d-h
SAH-HR2-L-PEG4-TC	DISGINASVVNIQKEIDRLNE8AKNLNEXLIDLQELG(K*)	Ac-	L	4	thiocholesterol	1d-h
SAH-HR2-M-PEG4-TC	DISGINASVVNIQKEIDRLNEV8KNLNESXIDLQELG(K*)	Ac-	M	4	thiocholesterol	1d-h
SAH-HR2-N-PEG4-TC	DISGINASVVNIQKEIDRLNEVA8NLNESLXDLQELG(K*)	Ac-	N	4	thiocholesterol	1d-h
SAH-HR2-O-PEG4-TC	DISGINASVVNIQKEIDRLNEVAK8LNESLIXLQELG(K*)	Ac-	O	4	thiocholesterol	1d-h
SAH-HR2-D-PEG0-TC	DISGINASVVNI8KEIDRLXEVAKNLNESLIDLQELG(K*)	Ac-	D	0	thiocholesterol	2a-c
SAH-HR2-D-PEG3-TC	DISGINASVVNI8KEIDRLXEVAKNLNESLIDLQELG(K*)	Ac-	D	3	thiocholesterol	2a-c
SAH-HR2-D-PEG5-TC	DISGINASVVNI8KEIDRLXEVAKNLNESLIDLQELG(K*)	Ac-	D	5	thiocholesterol	2a-c
SAH-HR2-D-PEG6-TC	DISGINASVVNI8KEIDRLXEVAKNLNESLIDLQELG(K*)	Ac-	D	6	thiocholesterol	2a-c
SAH-HR2-D-PEG7-TC	DISGINASVVNI8KEIDRLXEVAKNLNESLIDLQELG(K*)	Ac-	D	7	thiocholesterol	2a-c
SAH-HR2-D-PEG8-TC	DISGINASVVNI8KEIDRLXEVAKNLNESLIDLQELG(K*)	Ac-	D	8	thiocholesterol	2a-c
SAH-HR2-D-PEG10-TC	DISGINASVVNI8KEIDRLXEVAKNLNESLIDLQELG(K*)	Ac-	D	10	thiocholesterol	2a-c
SAH-HR2-D-PEG12-TC	DISGINASVVNI8KEIDRLXEVAKNLNESLIDLQELG(K*)	Ac-	D	12	thiocholesterol	2a-c
SAH-HR2-D-PEG16-TC	DISGINASVVNI8KEIDRLXEVAKNLNESLIDLQELG(K*)	Ac-	D	14	thiocholesterol	2a-c
SAH-HR2-D-PEG16-TC	DISGINASVVNI8KEIDRLXEVAKNLNESLIDLQELG(K*)	Ac-	D	16	thiocholesterol	2a-c
SAH-HR2-D-PEG20-TC	DISGINASVVNI8KEIDRLXEVAKNLNESLIDLQELG(K*)	Ac-	D	20	thiocholesterol	2a-c
RQ-01	DISGINASVVNI8KEIDRLXEVAKNLNESLIDLQELG(K*)	Ac-	D	8	cholesterol	2d-f, 2i, 3b-i, 4, 5, S4a-b, S5a-b, S7-9
RQ-01	DISGINASVVNI8KEIDRLXEVAKNLNESLIDLQELG(K*)	Btn-PEG$_2$-	D	8	cholesterol	3a
RQ-01 (-lipid)	DISGINASVVNI8KEIDRLXEVAKNLNESLIDLQELG(K*)	Btn-PEG$_2$-	D	8	-	3a
RQ-01 (-linker-lipid)	DISGINASVVNI8KEIDRLXEVAKNLNESLIDLQELG(K*)	Btn-PEG$_2$-	D	-	-	3a, S6
RQ-01 (-lipid)	DISGINASVVNI8KEIDRLXEVAKNLNESLIDLQELG(K*)	Ac-	D	8	-	3b
RQ-01 (-linker-lipid)	DISGINASVVNI8KEIDRLXEVAKNLNESLIDLQELG(K*)	Ac-	D	-	-	3b
SP4C	DISGINASVVNIQKEIDRLNEVAKNLNESLIDLQELGSGSGC	-	-	4	cholesterol	3c-i, S7
HR2 (aa 1168-1205)	DISGINASVVNIQKEIDRLNEVAKNLNESLIDLQELG(K*)	Ac-	-	-	-	S2, S6
RQ-01	DISGINASVVNI8KEIDRLXEVAKNLNESLIDLQELG(K*)	Cy5.5-βAla-	D	8	cholesterol	S4c
	**RSV-F (488-516)**					
HR2-PEG4-TC (RSV-F)	FDASISQVNEKINQSLAFIRKSDELLHNV(K*)	Ac-		4	thiocholesterol	6d
SAH-HR2-A-PEG4-TC	8DASISQXNEKINQSLAFIRKSDELLHNV(K*)	Ac-	A	4	thiocholesterol	6d
SAH-HR2-B-PEG4-TC	F8ASISQVXEKINQSLAFIRKSDELLHNV(K*)	Ac-	B	4	thiocholesterol	6d
SAH-HR2-C-PEG4-TC	FD8SISQVNXKINQSLAFIRKSDELLHNV(K*)	Ac-	C	4	thiocholesterol	6d
SAH-HR2-D-PEG4-TC	FDA8ISQVNEXINQSLAFIRKSDELLHNV(K*)	Ac-	D	4	thiocholesterol	6d
SAH-HR2-E-PEG4-TC	FDAS8SQVNEKXNQSLAFIRKSDELLHNV(K*)	Ac-	E	4	thiocholesterol	6d
SAH-HR2-F-PEG4-TC	FDASI8QVNEKIXQSLAFIRKSDELLHNV(K*)	Ac-	F	4	thiocholesterol	6d
SAH-HR2-G-PEG4-TC	FDASIS8VNEKINXSLAFIRKSDELLHNV(K*)	Ac-	G	4	thiocholesterol	6d
SAH-HR2-H-PEG4-TC	FDASISQ8NEKINQXLAFIRKSDELLHNV(K*)	Ac-	H	4	thiocholesterol	6d
SAH-HR2-I-PEG4-TC	FDASISQV8EKINQSXAFIRKSDELLHNV(K*)	Ac-	I	4	thiocholesterol	6d
SAH-HR2-J-PEG4-TC	FDASISQVN8KINQSLXFIRKSDELLHNV(K*)	Ac-	J	4	thiocholesterol	6d
SAH-HR2-K-PEG4-TC	FDASISQVNE8INQSLAXIRKSDELLHNV(K*)	Ac-	K	4	thiocholesterol	6d
SAH-HR2-L-PEG4-TC	FDASISQVNEK8NQSLAFXRKSDELLHNV(K*)	Ac-	L	4	thiocholesterol	6d
SAH-HR2-M-PEG4-TC	FDASISQVNEKI8QSLAFIXKSDELLHNV(K*)	Ac-	M	4	thiocholesterol	6d
SAH-HR2-N-PEG4-TC	FDASISQVNEKIN8SLAFIRXSDELLHNV(K*)	Ac-	N	4	thiocholesterol	6d
SAH-HR2-O-PEG4-TC	FDASISQVNEKINQ8LAFIRKXDELLHNV(K*)	Ac-	O	4	thiocholesterol	6d
SAH-HR2-P-PEG4-TC	FDASISQVNEKINQS8AFIRKSXELLHNV(K*)	Ac-	P	4	thiocholesterol	6d
SAH-HR2-Q-PEG4-TC	FDASISQVNEKINQSL8FIRKSDXLLHNV(K*)	Ac-	Q	4	thiocholesterol	6d
SAH-HR2-R-PEG4-TC	FDASISQVNEKINQSLA8IRKSDEXLHNV(K*)	Ac-	R	4	thiocholesterol	6d
SAH-HR2-S-PEG4-TC	FDASISQVNEKINQSLAF8RKSDELXHNV(K*)	Ac-	S	4	thiocholesterol	6d
SAH-HR2-T-PEG4-TC	FDASISQVNEKINQSLAFI8KSDELLXNV(K*)	Ac-	T	4	thiocholesterol	6d
SAH-HR2-U-PEG4-TC	FDASISQVNEKINQSLAFIR8SDELLHXV(K*)	Ac-	U	4	thiocholesterol	6d
SAH-HR2-V-PEG4-TC	FDASISQVNEKINQSLAFIRK8DELLHNX(K*)	Ac-	V	4	thiocholesterol	6d
SLI-RSV	FDASISQVNEKINQSL8FIRKSDXLLHNV(K*)	Ac-	Q	16	thiocholesterol	6g, S5c
	**EBOV-GP (600-631)**					
HR2-PEG4-TC (EBOV-GP)	TCHILGPDCAIEPHDWTKNITDKIDQIIHDFV(K*)	Ac-		4	thiocholesterol	6e
SAH-HR2-A-PEG4-TC	TCHILGPDCAIEPHDW8KNITDKXDQIIHDFV(K*)	Ac-	A	4	thiocholesterol	6e
SAH-HR2-B-PEG4-TC	TCHILGPDCAIEPHDWT8NITDKIXQIIHDFV(K*)	Ac-	B	4	thiocholesterol	6e
SAH-HR2-C-PEG4-TC	TCHILGPDCAIEPHDWTK8ITDKIDXIIHDFV(K*)	Ac-	C	4	thiocholesterol	6e
SAH-HR2-D-PEG4-TC	TCHILGPDCAIEPHDWTKN8TDKIDQXIHDFV(K*)	Ac-	D	4	thiocholesterol	6e
SAH-HR2-E-PEG4-TC	TCHILGPDCAIEPHDWTKNI8DKIDQIXHDFV(K*)	Ac-	E	4	thiocholesterol	6e
SAH-HR2-F-PEG4-TC	TCHILGPDCAIEPHDWTKNIT8KIDQIIXDFV(K*)	Ac-	F	4	thiocholesterol	6e
SAH-HR2-G-PEG4-TC	TCHILGPDCAIEPHDWTKNITD8IDQIIHXFV(K*)	Ac-	G	4	thiocholesterol	6e
SAH-HR2-H-PEG4-TC	TCHILGPDCAIEPHDWTKNITDK8DQIIHDXV(K*)	Ac-	H	4	thiocholesterol	6e
SAH-HR2-I-PEG4-TC	TCHILGPDCAIEPHDWTKNITDKI8QIIHDFX(K*)	Ac-	I	4	thiocholesterol	6e
SLI-EBOV	TCHILGPDCAIEPHDWT8EITDKIXQIIHDFV(K*)	Ac-	B	4	thiocholesterol	6h, S5d
	**NIPV-F (455-484)**					
HR2-PEG4-TC (NIPV-F)	DISSQISSBNQSLQQSKDYIKEAQKILDTV(K*)	Ac-		4	thiocholesterol	6f
SAH-HR2-A-PEG4-TC	8ISSQISXBNQSLQQSKDYIKEAQKILDTV(K*)	Ac-	A	4	thiocholesterol	6f
SAH-HR2-B-PEG4-TC	D8SSQISSXNQSLQQSKDYIKEAQKILDTV(K*)	Ac-	B	4	thiocholesterol	6f
SAH-HR2-C-PEG4-TC	DI8SQISSBXQSLQQSKDYIKEAQKILDTV(K*)	Ac-	C	4	thiocholesterol	6f
SAH-HR2-D-PEG4-TC	DIS8QISSBNXSLQQSKDYIKEAQKILDTV(K*)	Ac-	D	4	thiocholesterol	6f
SAH-HR2-E-PEG4-TC	DISSQ8SSBNQSXQQSKDYIKEAQKILDTV(K*)	Ac-	E	4	thiocholesterol	6f
SAH-HR2-F-PEG4-TC	DISSQI8SBNQSLXQSKDYIKEAQKILDTV(K*)	Ac-	F	4	thiocholesterol	6f
SAH-HR2-G-PEG4-TC	DISSQIS8BNQSLQXSKDYIKEAQKILDTV(K*)	Ac-	G	4	thiocholesterol	6f
SAH-HR2-H-PEG4-TC	DISSQISS8NQSLQQXKDYIKEAQKILDTV(K*)	Ac-	H	4	thiocholesterol	6f
SAH-HR2-I-PEG4-TC	DISSQISSB8QSLQQSXDYIKEAQKILDTV(K*)	Ac-	I	4	thiocholesterol	6f
SAH-HR2-J-PEG4-TC	DISSQISSBN8SLQQSKXYIKEAQKILDTV(K*)	Ac-	J	4	thiocholesterol	6f
SAH-HR2-K-PEG4-TC	DISSQISSBNQ8LQQSKDXIKEAQKILDTV(K*)	Ac-	K	4	thiocholesterol	6f
SAH-HR2-L-PEG4-TC	DISSQISSBNQS8QQSKDYXKEAQKILDTV(K*)	Ac-	L	4	thiocholesterol	6f
SAH-HR2-M-PEG4-TC	DISSQISSBNQSL8QSKDYIXEAQKILDTV(K*)	Ac-	M	4	thiocholesterol	6f
SAH-HR2-N-PEG4-TC	DISSQISSBNQSLQ8SKDYIKXAQKILDTV(K*)	Ac-	N	4	thiocholesterol	6f
SAH-HR2-O-PEG4-TC	DISSQISSBNQSLQQ8KDYIKEXQKILDTV(K*)	Ac-	O	4	thiocholesterol	6f
SAH-HR2-P-PEG4-TC	DISSQISSBNQSLQQS8DYIKEAXKILDTV(K*)	Ac-	P	4	thiocholesterol	6f
SAH-HR2-Q-PEG4-TC	DISSQISSBNQSLQQSK8YIKEAQXILDTV(K*)	Ac-	Q	4	thiocholesterol	6f
SAH-HR2-R-PEG4-TC	DISSQISSBNQSLQQSKD8IKEAQKXLDTV(K*)	Ac-	R	4	thiocholesterol	6f
SAH-HR2-S-PEG4-TC	DISSQISSBNQSLQQSKDY8KEAQKIXDTV(K*)	Ac-	S	4	thiocholesterol	6f
SAH-HR2-T-PEG4-TC	DISSQISSBNQSLQQSKDYI8EAQKILXTV(K*)	Ac-	T	4	thiocholesterol	6f
SAH-HR2-U-PEG4-TC	DISSQISSBNQSLQQSKDYIK8AQKILDXV(K*)	Ac-	U	4	thiocholesterol	6f
SAH-HR2-V-PEG4-TC	DISSQISSBNQSLQQSKDYIKE8QKILDTX(K*)	Ac-	V	4	thiocholesterol	6f
SLI-NIPV	DISSQISSBNQSL8QSKDYIXEAQKILDTV(K*)	Ac-	M	4	thiocholesterol	6i, S5e

Bird et al. Table S1

Supplementary Table 1 Compositions of peptides generated for the study
