# Supplementary material for: A stapled lipopeptide platform for preventing and treating highly pathogenic viruses of pandemic potential
Source: Nat Commun. 2024 Jan 4;15:274. doi: 10.1038/s41467-023-44361-1 (PMC10766962; doi:10.1038/s41467-023-44361-1)
Supplement: Supplementary file 3 — Reporting Summary [file 41467_2023_44361_MOESM3_ESM.pdf]

nature portfolio

Corresponding author(s): Loren D. Walensky

Last updated by author(s): Dec 8, 2023

Reporting Summary

Nature Portfolio wishes to improve the reproducibility of the work that we publish. This form provides structure for consistency and transparency in reporting. For further information on Nature Portfolio policies, see our Editorial Policies and the Editorial Policy Checklist.

Statistics

For all statistical analyses, confirm that the following items are present in the figure legend, table legend, main text, or Methods section.

n/a	Confirmed	
☐	☒	The exact sample size (*n*) for each experimental group/condition, given as a discrete number and unit of measurement
☐	☒	A statement on whether measurements were taken from distinct samples or whether the same sample was measured repeatedly
☐	☒	The statistical test(s) used AND whether they are one- or two-sided<br>*Only common tests should be described solely by name; describe more complex techniques in the Methods section.*
☒	☐	A description of all covariates tested
☒	☐	A description of any assumptions or corrections, such as tests of normality and adjustment for multiple comparisons
☐	☒	A full description of the statistical parameters including central tendency (e.g. means) or other basic estimates (e.g. regression coefficient) AND variation (e.g. standard deviation) or associated estimates of uncertainty (e.g. confidence intervals)
☐	☒	For null hypothesis testing, the test statistic (e.g. *F*, *t*, *r*) with confidence intervals, effect sizes, degrees of freedom and *P* value noted<br>*Give P values as exact values whenever suitable.*
☒	☐	For Bayesian analysis, information on the choice of priors and Markov chain Monte Carlo settings
☒	☐	For hierarchical and complex designs, identification of the appropriate level for tests and full reporting of outcomes
☒	☐	Estimates of effect sizes (e.g. Cohen's *d*, Pearson's *r*), indicating how they were calculated

Our web collection on statistics for biologists contains articles on many of the points above.

Software and code

Policy information about availability of computer code

Data collection	High content imaging data for pseudovirus and live virus infectivity assays were collected on a Molecular Devices ImageXpress Micro and Biotek Cytation 1, respectively. AlphaScreen data were collected using an Envision microplate reader.
Data analysis	Raw data were processed with Microsoft Excel for Microsoft 365 MSO (Version 2310 Build 16.0.16924.20054) and all plots, curve fittings, and statistical analyses performed in GraphPad Prism Version 10.

For manuscripts utilizing custom algorithms or software that are central to the research but not yet described in published literature, software must be made available to editors and reviewers. We strongly encourage code deposition in a community repository (e.g. GitHub). See the Nature Portfolio guidelines for submitting code & software for further information.

Data

Policy information about availability of data

All manuscripts must include a data availability statement. This statement should provide the following information, where applicable:

- Accession codes, unique identifiers, or web links for publicly available datasets
- A description of any restrictions on data availability
- For clinical datasets or third party data, please ensure that the statement adheres to our policy

All data generated or analyzed for this study are included in this manuscript and its Supplementary Information, including a Source Data file. Protein Data Bank entries with identification numbers 7TIK, 1G2C, 1EBO, and 1WP7 were used in the course of this study.

Research involving human participants, their data, or biological material

Policy information about studies with human participants or human data. See also policy information about sex, gender (identity/presentation), and sexual orientation and race, ethnicity and racism.

Reporting on sex and gender	N/A
Reporting on race, ethnicity, or other socially relevant groupings	N/A
Population characteristics	N/A
Recruitment	N/A
Ethics oversight	N/A

Note that full information on the approval of the study protocol must also be provided in the manuscript.

Field-specific reporting

Please select the one below that is the best fit for your research. If you are not sure, read the appropriate sections before making your selection.

☒ Life sciences ☐ Behavioural & social sciences ☐ Ecological, evolutionary & environmental sciences

For a reference copy of the document with all sections, see nature.com/documents/nr-reporting-summary-flat.pdf

Life sciences study design

All studies must disclose on these points even when the disclosure is negative.

Sample size	All biochemical and cellular experiments were performed in n=3 to 10 technical replicates to ensure an accurate analysis of the mean and standard deviation of a measurement, and then repeated in at least biological duplicate using independent experimental preparations. The number of biological and technical replicates are indicated in the figure legends and were chosen to ensure reproducibility in accordance with standard operation procedures for the corresponding experiments. Sample size calculations were not performed for biochemical and cellular studies (N/A); in vivo efficacy studies in hamsters were powered to detect statistically significant differences among treatment arms (e.g., n=8 per arm).
Data exclusions	No data were excluded during analysis.
Replication	All experiments were reproducibly performed in at least biological duplicate.
Randomization	For animal studies, rodents of similar age and weight range were randomly assigned to the indicated treatment groups. Randomization was not applicable to biochemical and cellular experiments as there was no allocation into groups.
Blinding	All hamster lung specimens for histopathologic analyses and scoring were submitted to the pathologist in blinded fashion. Blinding was not applicable for biochemical, cellular, and animal metrics with objective/automated read outs (e.g., viability assay, HPLC quantitation, animal weight, viral titer).

Reporting for specific materials, systems and methods

We require information from authors about some types of materials, experimental systems and methods used in many studies. Here, indicate whether each material, system or method listed is relevant to your study. If you are not sure if a list item applies to your research, read the appropriate section before selecting a response.

Materials & experimental systems

n/a	Involved in the study	
☐	☒	Antibodies
☐	☒	Eukaryotic cell lines
☒	☐	Palaeontology and archaeology
☐	☒	Animals and other organisms
☒	☐	Clinical data
☒	☐	Dual use research of concern
☒	☐	Plants

Methods

n/a	Involved in the study	
☒	☐	ChIP-seq
☒	☐	Flow cytometry
☒	☐	MRI-based neuroimaging

Antibodies

Antibodies used	Anti-SARS-CoV-2 nucleocapsid antibody (Sino Biological; RRID# AB_2827975)<br>Anti-EBOV GP antibody (IBT Bioservices; RRID# AB_2754983)<br>Anti-Ig secondary antibody (Alexa Fluor 488, Thermo Fisher Scientific; RRID# AB_2534088, AB_2576217)
Validation	All antibodies were validated by the manufacturers to interact with the species of proteins used in this study as referenced below.<br>1. Anti-SARS-CoV-2 nucleocapsid antibody (Sino Biological; RRID# AB_2827975): https://cdn1.sinobiological.com/reagent/40143-R004.pdf<br>2. Anti-EBOV GP antibody (IBT Bioservices; RRID# AB_2754983): https://www.ibtbioservices.com/wp-content/uploads/2019/04/Cat0201-020Lot1709001.pdf<br>3. Anti-Ig secondary antibody (Alexa Fluor 488, Thermo Fisher Scientific; RRID# AB_2534088, AB_2576217): https://www.thermofisher.com/antibody/product/Goat-anti-Mouse-IgG-H-L-Highly-Cross-Adsorbed-Secondary-Antibody-Polyclonal/A-11029 and https://www.thermofisher.com/antibody/product/Goat-anti-Rabbit-IgG-H-L-Highly-Cross-Adsorbed-Secondary-Antibody-Polyclonal/A-11034

Eukaryotic cell lines

Policy information about cell lines and Sex and Gender in Research

Cell line source(s)	293T-hACE2 (Integral Molecular; C-HA101; female), A549 (ATCC; CCL-185; male), HeLa (ATCC; CCL-2; female)
Authentication	Authentication performed by supplier as referenced below:<br>1. 293T-hACE2 (Integral Molecular; C-HA101): https://www.integralmolecular.com/wp-content/uploads/2020/07/1C01v2-293T-hsACE2-Cell-Maintenance.pdf and https://www.integralmolecular.com/wp-content/uploads/2020/05/293T-hsACE2-Cell-Maintenance-1.pdf<br>2. A549 (ATCC; CCL-185; STR Profiling): https://www.atcc.org/products/ccl-185<br>3. HeLa (ATCC; CCL-2; STR Profiling): https://www.atcc.org/products/ccl-2
Mycoplasma contamination	Cell lines tested negative for mycoplasma (MycoAlert, Lonza Biologics)
Commonly misidentified lines<br>(See ICLAC register)	No commonly misidentified cell lines were used in this study. All cell lines used in this study were purchased directly from the vendor, with authentication performed by the commercial supplier as indicated above.

Animals and other research organisms

Policy information about studies involving animals; ARRIVE guidelines recommended for reporting animal research, and Sex and Gender in Research

Laboratory animals	For PK analyses, male C57BL/6 mice, aged 6-7 weeks, and male Syrian hamsters (strain code 049), aged 8-9 weeks, were obtained from Charles River Labs. For in vivo efficacy testing, male and female Syrian hamsters (strain code 049) were obtained from Charles River labs. For the infection study, male and female hamsters were 64 days old with an average weight of 140 g; for the transmission study, male hamsters were 65 days old with an average weight of 148 g. Housing conditions for mice and hamsters were: 12 hour light/dark cycle, ambient temperature 70±2 °F, 50% humidity.
Wild animals	No wild animals were used in the study.
Reporting on sex	PK analyses were performed using male mice and hamsters, with overall rodent count for PK studies of 86. The treatment study was performed with an equal number of male and female hamsters (n=4 male and 4 female per arm) as delineated in the Source Data file. The study was designed for the data to be analyzed in aggregate, as powered to detect statistically significant differences among treatment arms (e.g., n=8 per arm). The transmission study was performed using only male hamsters to minimize fighting when animals were mixed. The overall rodent count was 48 for the treatment study (24 per sex) and 36 for the transmission study.
Field-collected samples	No field collected samples were used in this study.
Ethics oversight	The murine PK study was conducted under approved IACUC protocol VAS-103 at ATP Research & Development (Branford, CT). PK analysis and in vivo efficacy testing in hamsters were conducted under Colorado State University's approved IACUC protocol #1035 and IBC protocol #20-029B.

Note that full information on the approval of the study protocol must also be provided in the manuscript.

Plants

Seed stocks	N/A
Novel plant genotypes	N/A
Authentication	N/A
